# Blocking ETV6/RUNX1-induced MDM2 overexpression by Nutlin-3 reactivates p53 signaling in childhood leukemia

**DOI:** 10.1038/leu.2013.345

**Published:** 2013-12-13

**Authors:** U Kaindl, M Morak, C Portsmouth, A Mecklenbräuker, M Kauer, M Zeginigg, A Attarbaschi, O A Haas, R Panzer-Grümayer

**Affiliations:** 1 St Anna Kinderkrebsforschung, Children's Cancer Research Institute, Vienna, Austria; 2 St Anna Kinderspital, Medical University Vienna, Vienna, Austria

**Keywords:** ETV6/RUNX1-positive leukemia, MDM2, p53 pathway, Nutlin-3, ETV6/RUNX1 targets

## Abstract

*ETV6/RUNX1* (*E/R*) is the most common fusion gene in childhood acute lymphoblastic leukemia. It is responsible for the initiation of leukemia but also indispensable for disease maintenance and propagation, although its function in these latter processes is less clear. We therefore investigated the effects of the perceived p53 pathway alterations in model cell lines and primary leukemias and, in particular, how E/R upregulates MDM2, the predominant negative regulator of p53. We found that E/R transactivates MDM2 in both p53^+/+^ and p53^−/−^ HCT116 cells by binding to promoter-inherent RUNX1 motifs, which indicates that this activation occurs in a direct and p53-independent manner. Treatment of *E/R*-positive leukemic cell lines with Nutlin-3, a small molecule that inhibits the MDM2/p53 interaction, arrests their cell cycle and induces apoptosis. These phenomena concur with a p53-induced expression of p21, pro-apoptotic BAX and PUMA, as well as caspase 3 activation and poly ADP-ribose polymerase cleavage. The addition of DNA-damaging and p53-activating chemotherapeutic drugs intensifies apoptosis. Moreover, Nutlin-3 exposure leads to an analogous p53 accumulation and apoptotic surge in *E/R*-positive primary leukemic cells. Our findings clarify the role of p53 signaling in *E/R*-positive leukemias and outline the potential basis for its therapeutic exploitation in this setting.

## Introduction

With an incidence of approximately 25% *ETV6/RUNX1 (E/R)* is the most frequent fusion gene in childhood B-cell precursor leukemia.^1^ The encoded chimeric protein retains the N-terminal region of ETV6 and almost the entire RUNX1 protein including the DNA-binding domain.^2^ Despite the lack of high-risk features, up to 20% of affected children that are treated according to Berlin-Frankfurt-Münster-based protocols experience predominantly late relapses.^3^ In contrast to commonly expressed opinions, these relapses are often associated with drug resistance and poor outcome.^1, 4, 5^

There is ample evidence that the *E/R* gene fusion is most likely the initiating event in leukemia development. Although it provides the affected cells with an essential survival advantage, they nevertheless require additional mutations to evolve into overt leukemia.^2, 6, 7, 8, 9, 10^ Among the many possible mechanisms through which E/R fosters the survival of affected cells is the upregulation of survivin, which was recently identified by a small interfering RNA-mediated fusion gene knock-down (KD).^6, 7^ To better understand the transcriptional regulation and regulatory programs that E/R imposes on the leukemic cells, we also generated a lentiviral short hairpin RNA-carrying system to KD the chimeric protein in *E/R*-harboring leukemic cell lines.^11, 12^ These experiments facilitated the identification of the *MDM2* gene, the predominant negative regulator of p53 as a distinctively regulated gene. We therefore considered its upregulation of special relevance to the survival of these fusion gene-harboring leukemias.

*TP53* has the central role in the regulation of cell cycle, apoptosis, DNA repair and senescence and thereby acts as a gatekeeper of genomic integrity. It is activated by various stress signals and, in response, turns off proliferation by arresting the cell cycle to enable the appropriate repair of damaged DNA.^13, 14^ In case this fails, p53 also triggers apoptosis and senescence. p53 is precisely controlled by MDM2, which is an E3 ubiquitin ligase that targets p53 for ubiquitin-dependent degradation and therefore functions as a crucial negative regulator. In a feedback loop, p53 activates MDM2, which in turn inactivates p53 to prevent prolonged activation of p53. Thus, under physiological conditions these two proteins regulate each other in a dynamic manner and any imbalances result in a functional disturbance whose outcome heavily depends on the type and state of the affected cell.^14^

In consideration of its critical role as tumor suppressor, it is not surprising that *TP53* is mutated in approximately 50% of all cancers, and functionally silenced in many others.^15^ However, with an overall frequency below 5% at diagnosis and only up to 12% at relapse, *TP53* mutations are uncommon in acute lymphoblastic leukemia (ALL) and particularly scarce in childhood cases.^16, 17^ These rates also apply to the *E/R*-positive subgroup.^16^ So far, the possibility that this pathway can also be impaired by pure regulatory mechanisms, which do not involve or require mutational *TP53* alterations, has not yet been addressed systematically for this particular entity.

Our recent observations are of interest in this context, as they provided the first clues that this might indeed be the case. They indicated that an E/R-induced *MDM2* overexpression is the central and essential silencing factor of the p53 pathway.^12^ In this study, we therefore investigated how the presence of an *E/R* fusion gene might cause *MDM2* overexpression and how it impedes p53 signaling.

## Materials and methods

### Cell culture

JD Rowley (University of Chicago, IL, USA) kindly provided the *E/R*-positive B-cell precursor ALL cell lines AT-2 (ref. 18) and UoCB6 (ref. 19). The third *E/R*-positive leukemic cell line REH was obtained from the DSMZ (Braunschweig, Germany). All three cell lines lack genetic alterations of *TP53* and *MDM2* but harbor homozygous *CDKN2A* deletions as confirmed by single-nucleotide polymorphism arrays and fluorescence *in situ* hybridization. HCT116 p53^+/+^ and p53^−/−^ cell lines were kindly provided by B Vogelstein (Johns Hopkins University, Baltimore, MD, USA). Mouse putative pro-B *E/R*-expressing Ba/F3 clones and empty vector controls were established and cultured, as described previously.^6, 20^

Primary leukemic cells were obtained from bone marrow aspirations of children with ALL that were enrolled in the Austrian ALL-Berlin-Frankfurt-Münster 2000 protocol. The ethical committees of the Children's Cancer Research Institute and the St Anna Kinderspital approved this study. In accordance with the Declaration of Helsinki, we obtained written informed consent from the patients' parents for using spare material in this study.

### Quantitative reverse transcription-PCR

Total RNA was extracted using TRI Reagent (MRC Inc., Cincinnati, OH, USA) and used for complementary DNA synthesis as described previously.^11^ Human *MDM2* transcripts, *ABL* and *GUS* as endogenous controls, were quantified by TaqMan qRT-PCR using published primer probe combinations.^12^

### Cell cycle, viability and apoptosis assays

The cell cycle distribution was assessed with the Cycletest Plus DNA Reagent Kit (Becton Dickinson, Franklin Lakes, NJ, USA) according to the manufacturer's recommendations. Cell viability was determined by 3-(4,5-dimethylthiazol-2-yl)-2,5-diphenyltetrazoliumbromid colorimetric assay (Sigma-Aldrich, St Louis, MO, USA). The proportion of apoptotic cells was determined by flow cytometry using annexin V/propidium iodide and cleaved caspase 3 stainings. All assays were performed as described previously.^11^

### Western blot analysis

Cells were lysed, resolved and transferred as reported previously, using 60 μg of total protein.^11^ The primary antibodies used were: anti-MYC antibody (9E10), anti-glyceraldehyde 3-phosphate dehydrogenase antibody (6C5) and anti-p53 antibody (DO-1) (Santa Cruz Biotechnology Inc., Santa Cruz, CA, USA); anti-MDM2 antibody (OP143) and anti-p21 antibody (OP64) (Calbiochem, San Diego, CA, USA); anti-BAX (#2774) and anti-PUMA antibodies (#4976) (Cell Signaling Technology Inc., Danvers, MA, USA); anti-V5-horseradish peroxidase antibody (Invitrogen, Carlsbad, CA, USA) and anti-poly ADP-ribose polymerase antibody (C210) (Becton Dickinson). Secondary antibodies were horseradish peroxidase- or infrared dye-labeled (Bio-Rad, Hercules, CA, USA and LI-COR Biosciences, Lincoln, NE, USA, respectively) and proteins were visualized either with an enhanced chemiluminescence detection system (Thermo Scientific, Waltham, MA, USA) or membranes were scanned with the Odyssey Infrared Imaging System (LI-COR Biosciences), respectively.

### Quantification of intracellular proteins by flow cytometry

For detection of p53 protein, cells were fixed and permeabilized with the FoxP3 Staining Buffer Set (Biosience, Inc., San Diego, CA, USA) according to the manufacturer's recommendation. For cleaved caspase-3 staining, cells were fixed with 2% paraformaldehyde, permeabilized with 100% ice-cold methanol and incubated with mouse anti-p53 phycoerythrin antibody and rabbit anti-active caspase-3 phycoerythrin antibody (BD Pharmingen, Franklin Lakes, NJ, USA) for 30 min at room temperature. Samples were analyzed with a FACS Calibur flow cytometer (Becton Dickinson) and FlowJo software (Tree Star, Ashland, OR, USA).

### Luciferase reporter assays

Luciferase assays were performed using the MDM2 reporter construct (hdm2luc01) containing a 895-bp fragment of the P2 promoter region (kindly provided by JP Blaydes, University of Southampton, UK).^21^ This construct was used to transiently transfect HCT116 cells together with E/R (or empty vector as control), core binding factor β and p300 to enhance DNA binding, and pRL-TK Renilla (Promega, Madison, WI, USA) as transfection control, as described in detail elsewhere.^6, 20^ Cells were lysed 48 h after transfection and luciferase activities were measured using the Dual-Luciferase Reporter Assay System (Promega).

### Chromatin immunoprecipitation (ChIP)

ChIP was performed using HEK 293T, which stably expressed either E/R, a runt homology domain deleted ΔRHD-E/R mutant construct (kindly provided by O Williams, UK), or an empty vector, all tagged with a C-terminal V5. Two *E/R*-expressing HEK 293T clones were chosen (#21 with high and #26 with low amounts of fusion protein) to show dose dependency. In all, 2.5 × 10^7^ cells were used for each immunoprecipitation sample. Cells were incubated with formaldehyde (1%) at room temperature for 10 min, unreacted formaldehyde was quenched by addition of glycine (0.125 M, final concentration) and washed in cold phosphate-buffered saline with protease inhibitors (Roche, Basel, Switzerland). Nuclear extracts were prepared in 0.5 ml ChIP lysis buffer for 15 min on ice. Chromatin was sonicated to an average fragment size of 500 bp and centrifuged to remove cell debris. Fifty microliter aliquots of supernatant containing the crosslinked chromatin was transferred to fresh microfuge tubes. Two of them were supplemented with 450 μl ChIP dilution buffer and 10% each were removed as input control; to the remaining samples the different antibodies (mouse normal IgG antibody (Santa Cruz Biotechnology Inc.) and mouse anti-V5 antibody (Life Technologies, Carlsbad, CA, USA) and the fully suspended protein A magnetic beads were added. Beads were separated on a magnetic stand and sequentially washed with various buffers (low-salt, high-salt, LiCl and TE buffers). Chromatin was eluted (elution buffer) and DNA crosslinks were reversed by adding 1 μl proteinase K (20 mg/ml) and incubation at 65 °C for 2 h. DNA was recovered using a PCR purification kit (Qiagen, Hilden, Germany) and assessed by real-time PCR. The composition of the buffers is provided in the Supplementary Information.

### SYBR green real-time PCR

To amplify the putative RUNX1-binding site in the MDM2 promoter SYBR green real-time PCR was performed. The reaction included 2 × Maxima SYBR Green/ROX qPCR MasterMix (Thermo Scientific), 0.4 μM primers and 2 μl of immunoprecipitated DNA as a template in a 25 μl reaction volume. Primer sequences for the human *MDM2* RUNX1 motif-positive site were: (forward) 5′-TCAAGTTCAGACACGTTCCGAA-3′ and (reverse) 5′-ACTAAAGCTACAAGCAAGTCGGTG-3′. Primer sequences for the human *MDM2* RUNX1-negative site were: (forward) 5′-TGATGGATATGTTTGCTGCAGG-3′ and (reverse) 5′-GTGCACCAACAGACTTTAATAACTTC-3′.

### Bioinformatics and statistical analysis

Bioinformatic analyses were performed in R statistical environment using Bioconductor packages.^22^ A detailed description of gene expression profiling is provided elsewhere.^12^ For the re-analysis of Den Boer's primary ALL data,^23^ CEL files were downloaded from the GEO database (GSE13425) and normalized using the gcrma algorithm.^24^ Detailed information on gene sets used for gene set enrichment analysis can be found in the Molecular Signatures Database (http://www.broad.mit.edu/gsea/msigdb/; Cambridge, USA). For all Affymetrix microarray data, probe sets with very low expression values were excluded (R package: ‘panp') and one probe set with the highest variance across all samples was chosen for each gene. Differentially expressed genes were determined using a moderated *t*-test in the R package ‘limma'.^25^ All *P*-values were corrected for multiple testing using the ‘Benjamini–Hochberg' correction method. Differences between *E/R*-positive and *E/R*-negative samples as well as between different drug treatments were assessed by the unpaired *t*-test with Welch's correction by using the Graph Pad Prism Software (GraphPad Software, Inc., La Jolla, CA, USA). Average levels were expressed as mean±s.d. Statistical significance was considered when *P*<0.05.

## Results

### E/R shuts down p53 transcription

**Figure 1:**
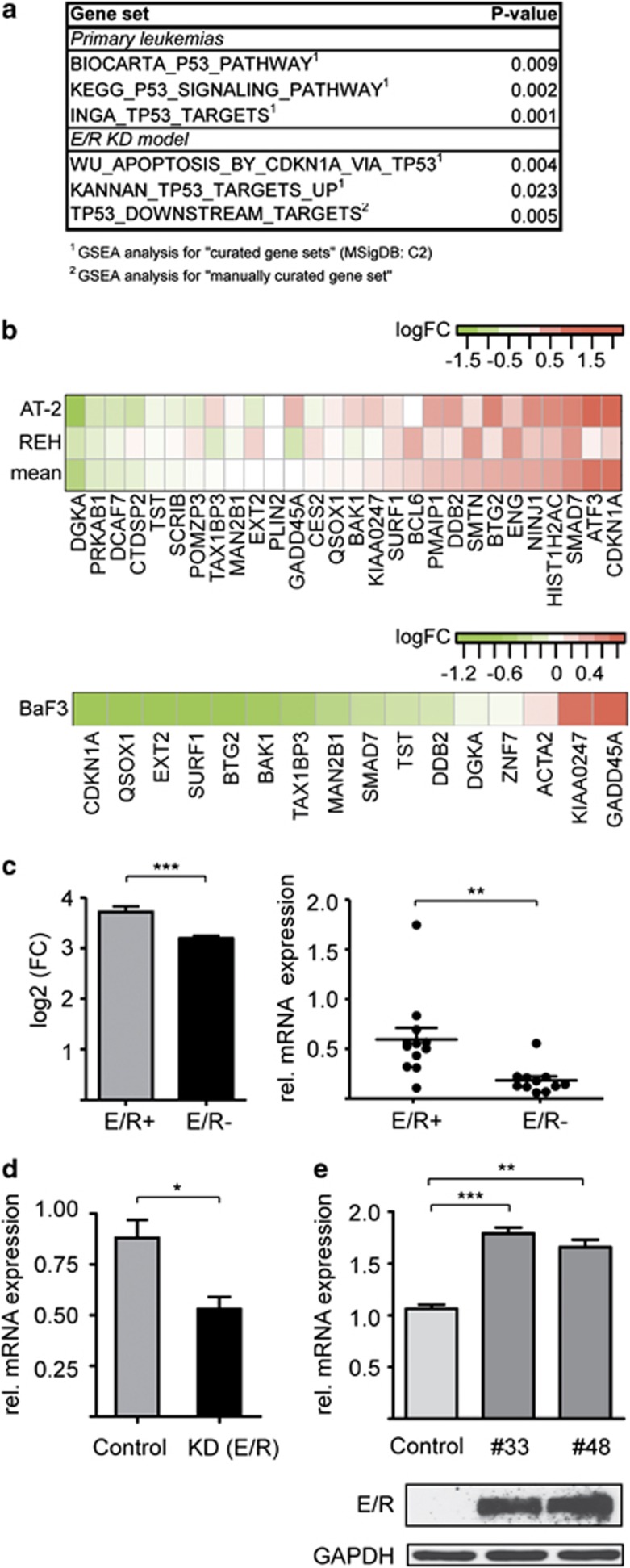
Deregulation of the p53 pathway by E/R. (**a**) Output from gene set enrichment analysis of primary leukemias and the E/R KD model. Depicted is a summary of significantly (*P*<0.05) de-regulated gene sets implicated in p53 signaling. (**b**) Heat map of the KANNAN_TP53_TARGETS_UP gene set, showing the de-repression of genes in the E/R KD model (top) and the repression of genes in E/R-expressing BaF3 cells (bottom). The differential expression is indicated by a color code, the respective scales are shown on top of each heat map. Positive log-ratios (log2-fold changes, log2 (FC)) are shown in red, negative log-ratios in green. (**c**) *MDM2* expression of microarray data^23^ from *E/R*-positive (E/R+) and *E/R*-negative (E/R–) primary B-cell precursor-ALL samples (left). Quantification of *MDM2* P2 transcript levels by quantitative reverse transcription-PCR (qRT-PCR). Shown is the relative expression of *MDM2* using *ABL* as a standard reference for normalization in *E/R*-positive (E/R+ *n*=12) and *E/R*-negative (E/R– *n*=11) primary ALL samples (right). (**d**) qRT-PCR analysis of MDM2 transcripts in E/R-suppressed REH cells and respective controls (non-targeting short hairpin RNA). Shown are *MDM2* levels determined in biological triplicates upon normalization. (**e**) *MDM2* transcripts (top) and E/R protein levels (bottom) of two different stably *E/R*-expressing Ba/F3 clones (#33 and #48). Anti-glyceraldehyde 3-phosphate dehydrogenase was used as loading control. Bars represent the mean±s.d. of three independent experiments. Welch's *t*-test, *P⩽0.05, **P⩽0.01, ***P⩽0.001.

On the basis of the previous suggestions that the E/R fusion protein is primarily responsible for the deranged p53 signaling, we aimed to identify differentially regulated genes by comparing microarray data of *E/R*-positive cell lines and primary leukemias with that of their fusion-negative B-cell precursor ALL counterparts. Gene set enrichment analysis revealed groups of genes that are involved in p53 signaling (Figure 1a). For additional comparisons, we also used the gene expression signature from two *E/R*-specific leukemic cell lines (REH and AT2) in which the endogenous fusion gene had been knocked down (Figure 1a).^12^ Two of the identified gene sets, the ‘KANNAN_TP53_TARGETS_UP' and ‘TP53_DOWNSTREAM_TARGETS', comprise mainly activated and proposed direct targets of p53.^26^ As shown in Figure 1b (upper part), *E/R* KD leads to the de-repression of p53 target genes. This pattern reverses when stably E/R-expressing Ba/F3 cells are used as a ‘first hit' model (*P*<0.05; Figure 1b, bottom).^6, 20^

We then generated a list of significantly and concordantly regulated genes from the pooled primary leukemia and KD model data set. We found that *MDM2* is not only one of the most prominent members of the 50 most highly upregulated genes (Supplementary Table 1), but that its expression is significantly more pronounced in *E/R*-positive than in *E/R*-negative ALL cases (Figure 1c left). As *MDM2* overexpression only suppresses p53 signaling in human cancers with a wt *TP53*, we explored whether this is also the case in *E/R*-positive leukemias.

### E/R upregulates *MDM2* in a p53-independent manner and by binding to a promoter-inherent RUNX1 motif

Quantification of *MDM2* transcripts in primary ALL samples confirms that the respective levels are significantly more abundant in *E/R*-positive (*n*=12) than in *E/R*-negative (*n*=11) cases (Figure 1c, right). In accordance, *MDM2* levels were lower in REH cells after *E/R* KD (Figure 1d) and higher in E/R-expressing Ba/F3 clones compared with parental cells (Figure 1e). These results strongly imply that E/R specifically attenuates the p53 pathway through upregulation of *MDM2*. Of note, none of the various leukemia expression profiles provided any evidence for a similar participation of *MDMX*, which is the second major negative regulator of p53,^13^ in E/R-positive cases.

**Figure 2:**
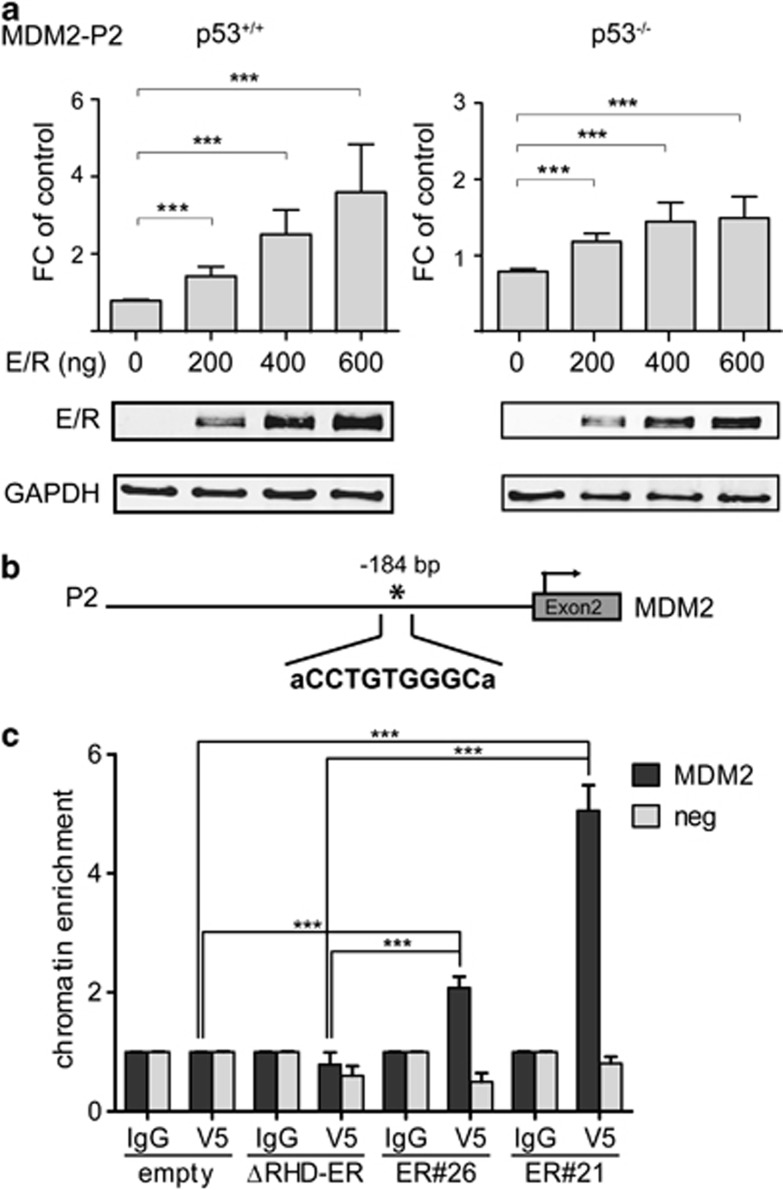
E/R transactivates MDM2 by binding to an endogenous RUNX1 motif. (**a**) Luciferase assays were performed using the *MDM2* P2 promoter construct to transfect HCT116 p53^+/+^ and p53^−/−^ cells together with increasing amounts of E/R expression vector (indicated at the x axis). Cells were co-transfected with core binding factor β to enhance binding and TK Renilla as transfection control. The total amount of DNA for each experiment was kept constant. Luciferase activity of pooled data of three independent experiments is depicted as fold-change (FC) of promoter activity of the empty vector. Welch's *t*-test, ***P⩽0.001. E/R expression by western blot analysis of the respective samples is shown at the bottom of the figure. (**b**) *In silico* search for putative *RUNX1*-binding sites in the MDM2 P2 promoter (895 bp) reporter construct used in (**a**) revealed one RUNX-binding motif 184-bp upstream of the transcription start (indicated by an asterisk). (**c**) ChIP assays at this putative RUNX1-binding site in HEK 293T cells stably expressing E/R (clones ER#21 and ER#26) and various controls (ΔRHD-ER; empty vector). Anti-V5 antibody was used for immunoprecipitation of E/R and mouse IgG as isotype control. MDM2 indicates the specific binding of E/R to the genomic region and ‘neg' is an MDM2 genomic region negative for E/R-binding sites. Welch's *t*-test, ***P⩽ 0.001. ER#26 vs ΔRHD-ER and empty vector are all *P*⩽ 0.001.

*MDM2* transcription is regulated by two promoters, which are activated in a p53-dependent and p53-independent manner, respectively.^27^ As in *E/R*-positive leukemias only the p53-dependent transcripts are differentially regulated, we investigated how E/R regulates the MDM2 P2 promoter as a function of p53 expression by performing luciferase reporter assays using the human isogenic p53^+/+^ and p53^−/−^ disparate HCT116 cell lines.^28^ We transiently transfected both of them with either an empty vector or various combinations and amounts of vector constructs that contained the P2 promoter sequences and the fusion transcript. E/R expression leads to an up to four-fold increase of *MDM2* promoter activity (Figure 2a) and transcription (Supplementary Figure 1) in wt p53, but also, albeit to a lesser extent, in p53^−/−^ cells (Figure 2a). These findings provide convincing evidence that E/R activates *MDM2* transcription in an autonomous and dose-dependent manner.

Therefore, we presumed that *MDM2* activation is most likely achieved through a direct promoter binding of the E/R protein. This notion is based on the fact that E/R acts as an aberrant transcription factor via its RHD in a cell context-dependent manner and either represses or directly activates RUNX1 targets, as recently reported for the erythropoietin receptor.^2, 20, 29, 30, 31, 32, 33^ Moreover, there are also good indications that p53 is not the only direct regulator of the MDM2 P2 promoter.^13^

To identify putative RUNX1-binding sites, we screened the MDM2 P2 promoter region *in silico* with Consite (http://asp.ii.uib.no:8090/cgi-bin/CONSITE/consite/), PATCH Search (http://www.gene-regulation.com/cgi-bin/pub/programs/patch/bin/patch.cgi) and JASPAR (http://jaspar.genereg.net/). All three programs identified one putative binding site (CCTGTGGGC) 184-bp upstream of the transcription start site in *MDM2* exon 2 (Figure 2b). To check whether E/R binds to this site, we performed ChIP assays with HEK 293T cells, which stably express either E/R-V5, ΔRHD E/R-V5 or contain an empty vector-V5. Cells were fixed, sonicated and incubated with either the anti-V5 antibody or the negative control antibody (mIgG). After isolation and purification of the immunoprecipitated DNA, SYBR green quantitative PCR was performed using primers that were specific for the putative RUNX1-binding site. The E/R-harboring cells were enriched ∼5-fold for the RUNX1 motif-positive MDM2 containing site proving that the chimeric protein occupies the respective chromatin area *in vivo* (Figure 2c). The corresponding *MDM2* expression is shown in the Supplementary Information (Supplementary Figure 2). These results provide first evidence that E/R directly binds to and positively regulates *MDM2* transcription from the P2 promoter.

### Inhibition of MDM2 reactivates the p53 pathway

Under physiological conditions, MDM2 has a short half-life and is a crucial negative modulator of p53 transcription. Pre-existing genetic lesions that increase or stabilize MDM2 expression thus reduce p53 levels and consequently abrogate p53 signaling.^13, 14^ As proteasomal degradation is not compromised in *E/R*-positive leukemic cell lines (data not shown), we tested whether Nutlin-3, a small molecule that disrupts the MDM2/p53 interaction, can reactivate p53 signaling.^13^

**Figure 3:**
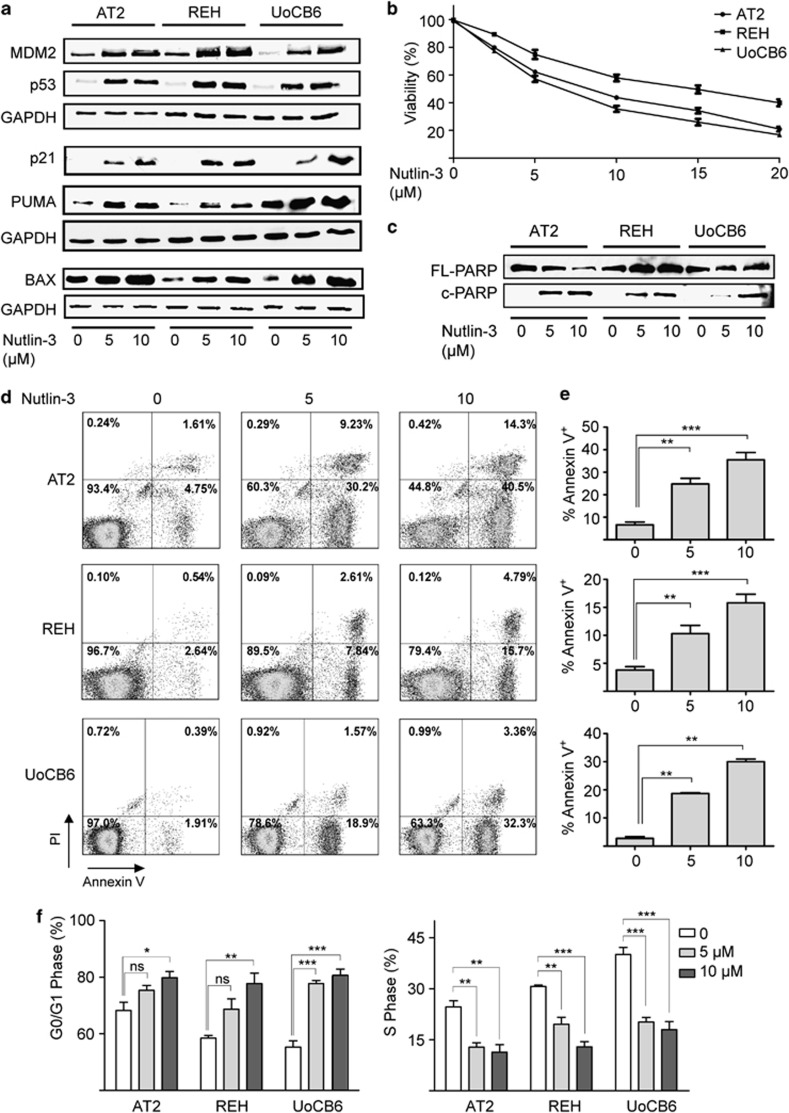
Reactivation of p53 by Nutlin-3 in *E/R*-expressing leukemic cell lines. (**a**) Western blot analysis of AT2, REH and UoCB6 cells confirms accumulation of p53 and induction of downstream targets MDM2, PUMA, BAX and p21 upon exposure to Nutlin-3. Glyceraldehyde 3-phosphate dehydrogenase (GAPDH) was used as loading control. Results are representative of three independent experiments. (**b**) Viability (3-(4,5-dimethylthiazol-2-yl)-2,5-diphenyltetrazoliumbromid) assays of cell lines upon exposure to Nutlin-3. Pooled data of three independent experiments are shown. (**c**-**e**) Apoptosis was assessed by western blot analysis for poly ADP-ribose polymerase cleavage (FL, full-length; c, cleaved) (shown in **c**), annexin-V/propidium iodide (PI) staining (representative histograms in **d**) and proportions of Annexin-V/PI+ cells (%) (**e**) of three independent experiments. (**f**) Distribution of cells in G0/G1 and S phase relative to carrier control upon exposure to Nutlin-3. Data are derived from at least three independent experiments. Welch's *t*-test, *P⩽0.05, **P⩽0.01, ***P⩽0.001. NS, not significant.

For this purpose, we exposed *E/R*-positive leukemic cell lines to various clinically meaningful concentrations (1–10 μM) of Nutlin-3. This treatment led to a substantial accumulation of the p53 protein as well as the expression of its direct targets p21, MDM2 and the pro-apoptotic BAX and PUMA proteins in all instances (Figure 3a). As the consequences of p53 reactivation depend on the specific cellular context,^34, 35^ we investigated how Nutlin-3 affects the cell cycle distribution as well as the viability of cells and their propensity to undergo apoptosis. As shown in Figures 3b–e, Nutlin-3 treatment significantly reduced viability and increased apoptosis in a dose-dependent manner. Consistent with the induction of p21 it also raised the proportion of cells in the G0/G1 and lowered that of cells in the S-phase (Figure 3f, Supplementary Figure 3). Collectively, these data demonstrate that Nutlin-3 restores p53 function in *E/R*-expressing leukemic cell lines and underscore the relevance of p53 pathway signaling alterations for the disease.

### Nutlin-3 enhances drug-induced apoptosis in *E/R*-positive primary leukemias

We also checked whether the addition of Nutlin-3 can potentiate the apoptosis-inducing effect of DNA-damaging and p53-activating chemotherapeutic drugs, such as daunorubicin, asparaginase and vincristine, which are currently used worldwide in all major childhood ALL treatment protocols.^1, 26^ The first two drugs activate the DNA damage checkpoint mainly by inducing DNA double- and/or single-strand breaks, whereas the latter by disrupting the mitotic spindle.

**Figure 4:**
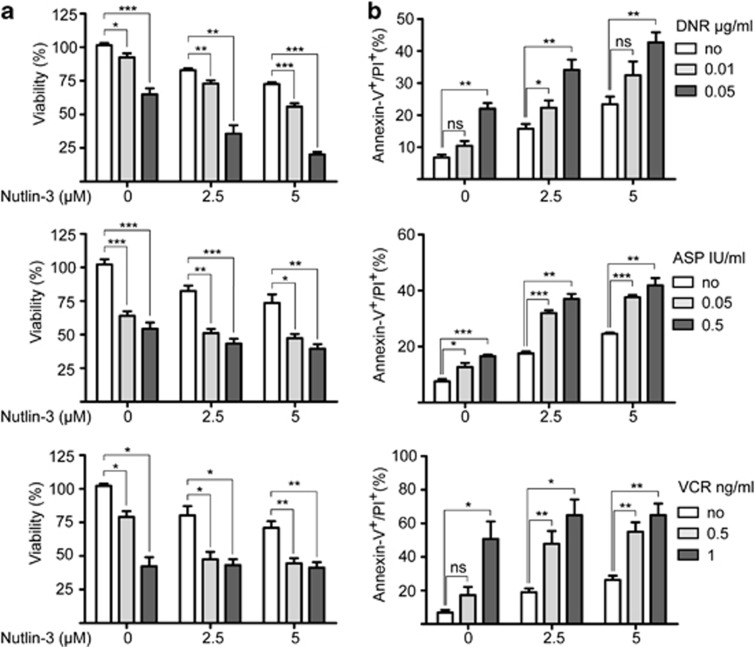
Nutlin-3 enhances effects of chemotherapeutic agents in *E/R*-expressing cell lines. REH cells were exposed to daunorubicin (DNR), vincristine (VCR) and asparaginase (ASP) alone and in combination with Nutlin-3. (**a**) Viability was determined by 3-(4,5-dimethylthiazol-2-yl)-2,5-diphenyltetrazoliumbromid assay and is indicated relative to carrier control. (**b**) Apoptotic fractions were measured by flow cytometry. The percentage of apoptotic cells (annexinV+/ propidium iodide (PI)+) is shown. Data were derived from at least three independent experiments. Welch's *t*-test, *P⩽0.05, **P⩽0.01, ***P⩽0.001. NS, not significant.

We exposed the *E/R*-positive REH and UoCB6 cell lines to low concentrations of Nutlin-3 (2.5 and 5 μM) together with the aforementioned drugs using established concentrations (daunorubicin, 0.01 and 0.05 μg/ml; asparaginase, 0.05 and 0.5 IU/ml; vincristine, 0.5 and 1 ng/ml).^36, 37, 38^ 3-(4,5-Dimethylthiazol-2-yl)-2,5-diphenyltetrazoliumbromid assays were performed 48 h after co-exposure with the chemotherapeutic agents and revealed a reduction of cell survival by up to 80% and increase of apoptosis by 42% (for daunorubicin and asparaginase) and 64% (for vincristine), whereas the comparable rates with Nutlin-3 alone were significantly lower (Figure 4 and Supplementary Figure 4).

**Figure 5:**
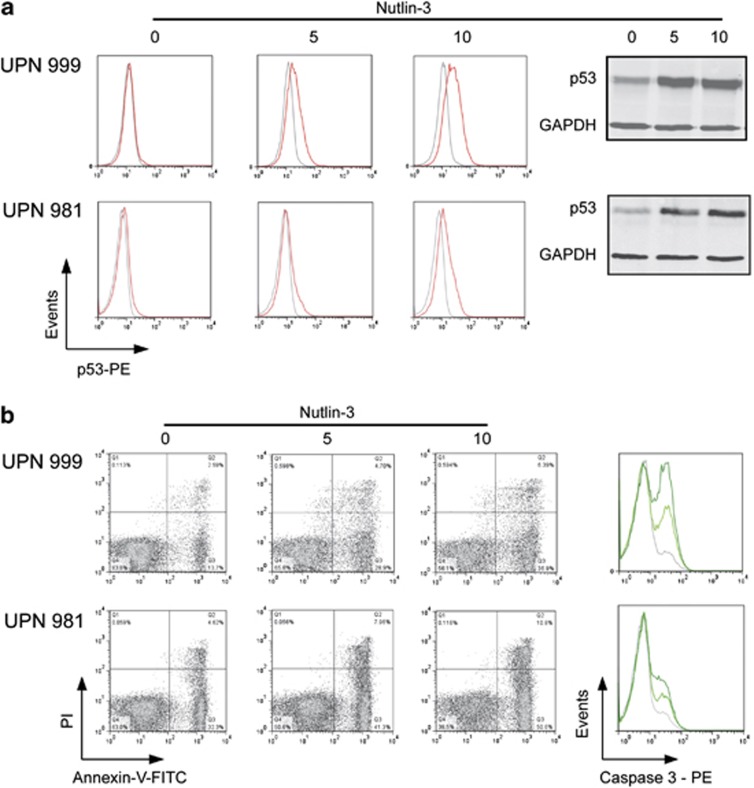
Nutlin-3 reactivates p53 and induces apoptosis in primary ALL samples. Representative examples of *E/R*-positive (UPN 999, top) and *E/R*-negative (UPN 981, bottom) primary leukemia samples upon exposure to Nutlin-3 are depicted. (**a**) Left part: flow cytometric analysis of p53 expression (red) and respective isotype control (gray); corresponding western blot using glyceraldehyde 3-phosphate dehydrogenase (GAPDH) as control (right). (**b**) Apoptosis was assessed by AnnexinV/propidium iodide (PI; left) and cleaved caspase 3 stainings (right); green lines, upon exposure to Nutlin-3 (light green, 5 μM; dark green, 10 μM concentration); gray lines, untreated control. PE, phycoerythrin.

To confirm that Nutlin-3 exerts a similar effect also on primary leukemias, we used *E/R*-positive samples with >90% blasts from children who were consecutively enrolled in the Austrian ALL-Berlin-Frankfurt-Münster 2000 protocol. Appropriate *E/R*-negative samples were used as control. As an initial assessment by western blot and flow cytometry yielded equal estimates of p53 protein levels (Figure 5a), we used only flow cytometry for the consecutive analyses. As observed in the cell lines, Nutlin-3 exposure increased p53 protein levels and the proportion of apoptotic cells in a dose-dependent manner in all samples (Figure 5b). In accordance with the generally higher MDM2 levels, however, also the apoptosis rates were significantly more pronounced in *E/R*-positive than in *E/R*-negative samples (Supplementary Figure 5).

## Discussion

The *E/R* fusion gene drives not only the leukemic transformation process but its sustained expression is also indispensable for the progression and maintenance of the disease.^11, 39^ Previous evidence has already indicated that suppression of p53 signaling is a crucial element in this development and that the chimeric E/R protein does probably directly deregulate this pathway, although the mode of its action has remained unexplored so far.^12^ Herein we now show, in various cellular model systems and primary leukemias, that the aberrant E/R transcription factor binds to the *MDM2* P2 promoter and consequently upregulates MDM2 in a direct and p53-independent manner. Nutlin-3, a specific inhibitor of the MDM2/p53 interaction, is able to relieve the ensuing p53 attenuation, which leads to cell cycle arrest and enhanced apoptosis in the affected cells.

The p53 signaling pathway is probably affected in the vast majority of all types of cancer in one way or the other. Its function can be disrupted either by various types of genetic alterations (as for instance, *TP53* mutations, *CDKN2A* deletions) or by a sustained expression of *MDM2*, the main negative p53 regulator. This latter mode of inactivation is also observed in *E/R*-positive leukemias. So far, the focus of interest in *MDM2* overexpression has been on drug-resistant childhood ALL cases, whereas its potential relevance in *E/R*-positive cases has been largely disregarded because of the comparably favorable outcome of these leukemias. However, *MDM2* overexpression seems to be a distinct and rather unique feature of *E/R*-positive ALL, because to this extent it is only found in this particular genetic subgroup. Our earlier functional analyses of leukemic cell lines, in which the *E/R* fusion gene had been knocked down, already implied that *MDM2* overexpression is the essential factor that abolishes p53 function.^12^ Moreover, no other possible activation routes, such as *MDM2* gene amplifications, are encountered in such cases.^4, 10, 40^ The only other conceivable route that might perhaps also lead to *MDM2* upregulation and consequently to the impairment of p53 signaling is the loss of p14^ARF^, a *CDKN2A*-encoded inhibitor of MDM2, which is deleted in up to 30% of *E/R*-positive cases.^4, 10, 41^

Our observation that E/R activates *MDM2* directly via binding to a RUNX1 motif in the P2 promoter region fits perfectly to its role as aberrant transcription factor. It adds evidence that E/R can regulate the respective RUNX1 target genes not only by repressing but also by activating them.^29^ This aspect was first noted by Neil's group and was based on data that were obtained in a murine fibroblast cell line.^31^ More recently, it was further corroborated in human *E/R*-positive cell lines and primary leukemic samples by demonstrating that E/R binds and activates the promoter of the erythropoietin receptor, whose atypical expression seems to be another characteristic feature of *E/R*-positive leukemias.^31, 32, 33^ On a broader and more general scale, the significance of this phenomenon is also supported by the results of our previous fusion gene KD experiment, in which it became evident that approximately half of all E/R-regulated genes are upregulated, a substantial number of which contain putative RUNX1-binding sites in their promoter regions.^12^ These RUNX1-binding motifs were verified by ChIP sequencing in material that derived from human hematopoietic stem and progenitor cells as well as megakaryocytes.^42, 43^

To study the effects of an impeded MDM2 function on p53, we exposed the respective cells to Nutlin-3, a small molecule inhibitor of the MDM2/p53 interaction. On the basis of its ability to reactivate p53 in malignancies with a wt *TP53* and because toxicity of this agent is negligible in normal cells, it is already being tested in clinical trials.^13, 44^ Previous studies have found that Nutlin-3 variably arrests the cell cycle and induces apoptosis in leukemic cell lines with a wt *TP53*, the extent of which depends on the level of MDM2 expression.^13^ In our experiments, however, Nutlin-3-induced apoptosis and merely a G0/G1 arrest but not, as reported previously in some cancers, also a G2/M arrest.^13^ Although we cannot rule out that cells arrested in G2/M did not accumulate and appear as a discernible fraction because they immediately underwent apoptosis, it seems more likely that an E/R-mediated repression of MAD2L1, together with the abrogation of the mitotic cell cycle control, interfered with the potential p53-mediated G2/M check point function.^20^

The E/R-induced upregulation of MDM2 in Ba/F3 murine pro-B cells indicates that this occurs merely as a function of the fusion gene early on during leukemia development and is found in the absence of secondary aberrations. In support of this view, the expression of *RUNX1-ETO*, a related *RUNX1* gene fusion that occurs in acute myeloid leukemias, also upregulates *MDM2* and thereby represses p53 signaling in hematopoietic stem cells.^45^ Moreover, treatment of these transduced cells with Nutlin-3 reversed the observed increased proliferation and self-renewal. Similarly, Nutlin-3 reduced proliferation in *E/R*-positive leukemic cells by cell cycle arrest and apoptosis, which was enhanced upon exposure to chemotherapeutic drugs. These effects were more pronounced in primary *E/R*-positive than *E/R*-negative cases, which again underlines that this phenomenon is a unique and inherent feature of this particular genetic subgroup. In this context, it is also of special interest that the activation of the phosphatidylinositol 3 kinase/Akt pathway promotes the phosphorylation of MDM2 and its localization to the nucleus where it binds to p53 and enhances ubiquitin-mediated degradation.^44, 46^ Of note, activation of the phosphatidylinositol 3 kinase/Akt pathway emerged also as one of the prominent features in our E/R fusion gene KD experiments.^11^

**Figure 6:**
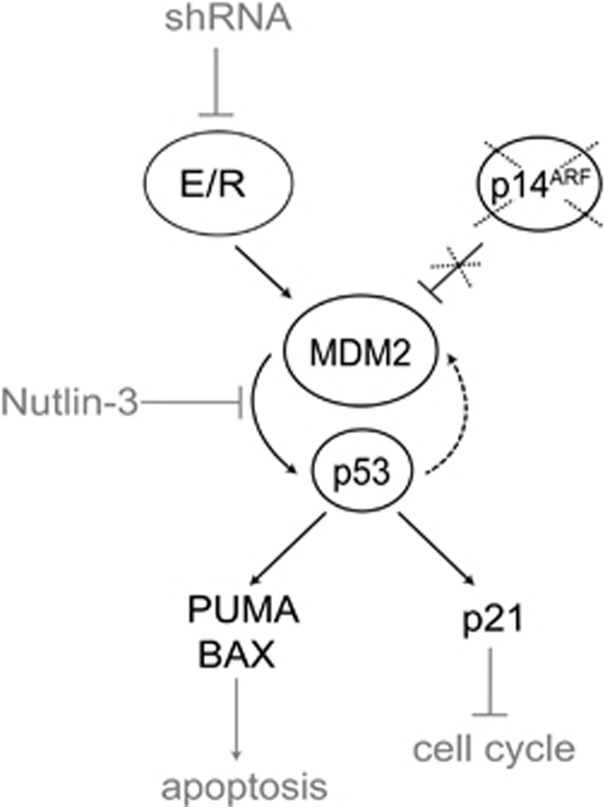
Model for p53 pathway repression by E/R. E/R inhibits p53 by upregulating MDM2 expression, a process that can be reversed by suppressing E/R with short hairpin RNA. Coexisting deletions of *CDKN2A*, which encodes the MDM2 inhibitor p14^ARF^, can further consolidate MDM2 expression and the ensuing effects on p53. Disrupting this inappropriate MDM2/p53 interaction with the small molecule inhibitor Nutlin-3 restores p53 function leading to upregulation of p21, BAX and PUMA and consequently arrests the cell cycle and induces apoptosis in E/R-positive cells.

On the basis of our results, we present a model that outlines how E/R impairs p53 function and promotes leukemia development and progression (Figure 6). In this scenario, E/R directly upregulates MDM2 and thereby represses p53, which is a prerequisite for the competitive survival advantage of the transformed cells. This unstable genomic state might also predispose the cells to the development of additional genetic lesions, such as *CDKN2A* deletions. The ensuing reduction of the encoded p14^ARF^ protein finally mitigates the p14^ARF^-mediated control of MDM2 activity and thereby further enhances the degradation of p53.

In conclusion, we have provided strong evidence that E/R abrogates p53 signaling through the direct induction of MDM2 expression, a cellular attribute that can be reversed *in vitro* by the inhibition of MDM2. Taken together, these finding suggest (1) that disruption of the p53 signaling pathway is an important mechanism, which promotes cell survival and probably also facilitates the acquisition of secondary aberrations and (2) that the pharmacological inhibition of the respective MDM2/p53 interaction with small molecules may provide new specific avenues for individualized therapeutic interventions in this group of patients.
