# Supplementary material for: Blocking ETV6/RUNX1-induced MDM2 overexpression by Nutlin-3 reactivates p53 signaling in childhood leukemia
Source: Leukemia. 2013 Dec 13;28(3):600–8. doi: 10.1038/leu.2013.345 (PMC3948158; doi:10.1038/leu.2013.345)
Supplement: Supplementary Information [file leu2013345x1.pdf]

SUPPLEMENTARY INFORMATION

Blocking ETV6/RUNX1-induced MDM2 overexpression by Nutlin-3 reactivates p53 signaling in childhood leukemia

Ulrike Kaindl[1,*], Maria Morak[1,*], Christine Portsmouth[1], Astrid Mecklenbräuker[1], Max Kauer[1], Marion Zeginigg[1], Andishe Attarbaschi[2], Oskar A. Haas[1,2], and Renate Panzer-Grümayer[1]

[1] Children´s Cancer Research Institute, St. Anna Kinderkrebsforschung, Vienna, Austria

[2] St. Anna Kinderspital, Medical University Vienna, Vienna, Austria

* These authors contributed equally to this work

Supplementary Methods

Cell culture

All cell lines were cultured as reported previously[1] and primary leukemic cells were resuspended in X-VIVO™ 10 Medium (Lonza, Visp, CH). Nutlin-3 was purchased from Calbiochem (San Diego, CA, USA), dissolved in DMSO and used at 2.5-10 µM concentrations. Daunorubicin, Vincristine (Pfizer, Inc., NY, USA) and L-Asparaginase (Medac GmbH, Wedel, Germany) were diluted in NaCl and used at indicated concentrations. HCT116 cells were transfected by Lipofectamine 2000 (Invitrogen) according to the manufacturer´s recommendations. E/R knock-down was performed as described previously [2] and RNA was isolated 2-3 weeks after lentiviral transduction and confirmation of chimeric protein depletion.

Quantitative reverse transcription polymerase chain reaction (qRT-PCR)

For qRT-PCR analysis of MDM2 transcripts, 2 µg of RNA was reverse transcribed using a mixture of oligo(dT)$_{15}$, random hexamer primers and M-MLV Reverse Transcriptase to generate cDNA. To amplify mouse MDM2 and GAPDH transcripts, the following primer/probe combinations were used: MDM2, (f) 5'-CTGTGTCTACCGAGGGTGCT-3'; (r) 5'-ATGTGCTGCTGCTTCTCGT-3'; (p) 5'-FAM-CGTTGGAGCGCAAAACGACA-TAMRA-3'; GAPDH, (f) 5'-TGTGTCCGTCGTGGATCTGA-3'; (r) 5'-CCTGCTTCACCACCTTCTTGAT-3'; (p) 5'-FAM-CCGCCTGGAGAAACCTGCCAAGTATG-TAMRA-3'. GAPDH was used as a standard reference for normalization. The quantitative PCRs were done in a total volume of 25 µl containing 12.5 µl TaqMan Universal PCR Master Mix (Applied Biosystems, Foster City, CA, USA), 100 nmol/l forward and reverse primers each and 50 nmol/l Taqman probe. The following cycling conditions were used: 95°C for 10', 95°C for 15'' and 58 - 63°C according to the different primer and probe combinations for 1'. The last two steps were repeated 50 times.

Western blot analysis

After the addition of 2x loading buffer samples were incubated at 95°C for 5 min. Sixty µg of protein were size separated on 10 - 15 % SDS PAGE, blotted on nitrocellulose transfer membrane (Whatman, GE Healthcare, Chalfont St. Giles, UK), blocked with 1x blocking reagent (Roche) in TBS and incubated with the respective antibody at 4°C overnight. Secondary antibodies were HRP- or (Biorad, Hercules, USA) infrared dye-labeled (LI-COR

Biosciences, Lincoln, NE) and proteins were visualized with enhanced chemiluminscence detection system (Thermo Scientific, Waltham, MA, USA) or membranes were scanned with Odyssey Infrared Imaging System (LI-COR Biosciences, NE, USA) respectively. All western blots shown are representative of at least three independent experiments.

ChIP buffers

ChIP lysis buffer: 1% SDS, 10 mM EDTA, 50 mM Tris-HCl pH 8.0 and protease inhibitors; ChIP dilution buffer: 0.01% SDS, 1% Triton-X 100, 1.2 mM EDTA, 16.7 mM Tris-HCl pH 8.0, 167 mM NaCl and protease inhibitors; Low-salt buffer: 0.1% SDS, 1% Triton X-100, 2 mM EDTA, 20 mM Tris-HCl pH 8.0, 150 mM NaCl; High-salt buffer: 0.1% SDS, 1% Triton X-100, 2mM EDTA, 20mM Tris-HCl pH 8.1, 500 mM NaCl; LiCl buffer: 0.25 M LiCl, 1% NP-40, 1% deoxycholate, 1 mM EDTA and 10 mM Tris-HCl pH 8.1; TE buffer: 10 mM Tris-HCl pH 8.0, 1 mM EDTA; Elution buffer: 1% SDS and 0.1 M $NaHCO_3$

Supplementary Table 1. Top 50 upregulated genes by E/R

	Gene symbol	Entrez Gene ID	E/R KD REH	E/R KD AT2	E/R KD mean	P-value	adj. P-value	ER+ vs ER-	P-value ER+ vs ER-	adj. P-value ER+ vs ER-
1	BMP2	650	-2.29	-0.98	-1.63	0.0006	0.0171	1.6556	0.00000	0.0000
2	DCHS1	8642	-1.24	-1.97	-1.60	0.0001	0.0059	1.0079	0.00000	0.0000
3	SEMA6A	57556	-1.55	-1.58	-1.56	0.0000	0.0033	0.3505	0.00036	0.0041
4	ANO1	55107	-1.93	-1.10	-1.52	0.0004	0.0126	0.4811	0.00000	0.0000
5	DRAM1	55332	-1.16	-1.16	-1.16	0.0000	0.0019	0.3776	0.00000	0.0000
6	MDM2	4193	-1.02	-1.21	-1.11	0.0000	0.0019	0.5817	0.00000	0.0000
7	TRIB1	10221	-0.34	-1.84	-1.09	0.0001	0.0047	1.0848	0.00000	0.0001
8	INPP5D	3635	-0.98	-1.07	-1.03	0.0009	0.0210	0.3949	0.00686	0.0411
9	HIST2H2BE	8349	-1.61	-0.21	-0.91	0.0008	0.0194	0.8964	0.00087	0.0081
10	FYB	2533	-0.68	-1.15	-0.91	0.0003	0.0117	0.6357	0.00000	0.0000
11	SOX11	6664	-1.14	-0.54	-0.84	0.0003	0.0107	2.2622	0.00000	0.0000
12	PVRIG	79037	0.43	-2.08	-0.82	0.0038	0.0466	0.5947	0.00116	0.0103
13	ARHGEF4	50649	-1.03	-0.59	-0.81	0.0002	0.0080	1.1283	0.00000	0.0000
14	TLE4	7091	-0.56	-1.01	-0.79	0.0021	0.0334	0.6286	0.00125	0.0108
15	KHDRBS3	10656	-0.39	-1.11	-0.75	0.0000	0.0040	0.9730	0.00001	0.0002
16	PRKAR2B	5577	-0.59	-0.89	-0.74	0.0000	0.0043	0.4625	0.00103	0.0093
17	B4GALT6	9331	-0.73	-0.71	-0.72	0.0000	0.0043	0.2224	0.00017	0.0022
18	PTPRK	5796	-0.70	-0.69	-0.70	0.0001	0.0049	0.5882	0.00379	0.0261
19	PIK3C3	5289	-0.46	-0.93	-0.69	0.0001	0.0068	0.8371	0.00000	0.0000
20	NDFIP1	80762	-1.07	-0.25	-0.66	0.0041	0.0482	0.4428	0.00049	0.0052
21	ZNF268	10795	-0.94	-0.36	-0.65	0.0006	0.0165	0.1535	0.00008	0.0012
22	SCARB1	949	-0.51	-0.77	-0.64	0.0015	0.0282	0.6792	0.00000	0.0000
23	RAB1A	5861	-0.75	-0.51	-0.63	0.0002	0.0104	0.6657	0.00006	0.0009
24	SLC35E3	55508	-0.44	-0.79	-0.62	0.0002	0.0083	0.6119	0.00133	0.0114
25	AKAP12	9590	-0.56	-0.56	-0.56	0.0010	0.0222	0.9180	0.00762	0.0440
26	BTBD3	22903	-0.48	-0.62	-0.55	0.0002	0.0100	1.0385	0.00000	0.0000
27	FAM134B	54463	-0.59	-0.50	-0.54	0.0027	0.0387	0.4522	0.00016	0.0021
28	TNFRSF21	27242	-0.33	-0.75	-0.54	0.0013	0.0257	2.4108	0.00000	0.0000
29	GPR125	166647	-0.76	-0.30	-0.53	0.0011	0.0238	0.5559	0.00000	0.0000
30	RGL1	23179	-0.48	-0.55	-0.52	0.0006	0.0160	0.7800	0.00007	0.0010
31	TLE3	7090	-0.14	-0.90	-0.52	0.0021	0.0336	1.6644	0.00000	0.0000
32	TSPAN9	10867	-0.69	-0.35	-0.52	0.0008	0.0196	0.4682	0.00002	0.0003
33	TBC1D9	23158	-0.53	-0.50	-0.52	0.0006	0.0160	1.2922	0.00000	0.0000
34	GNAQ	2776	-0.25	-0.78	-0.52	0.0010	0.0227	0.6355	0.00001	0.0003
35	MME	4311	-0.49	-0.49	-0.49	0.0029	0.0401	0.9211	0.00204	0.0160
36	EIF4A2	1974	-0.18	-0.78	-0.48	0.0004	0.0142	0.2684	0.00624	0.0382
37	KIAA0232	9778	-0.78	-0.16	-0.47	0.0037	0.0456	0.5108	0.00376	0.0259
38	ERO1LB	56605	-0.43	-0.49	-0.46	0.0021	0.0334	0.2934	0.00708	0.0420
39	GNG11	2791	-0.40	-0.52	-0.46	0.0009	0.0206	2.9448	0.00000	0.0000
40	GRAMD1B	57476	-0.54	-0.36	-0.45	0.0015	0.0276	0.3283	0.00007	0.0011
41	OTUD4	54726	-0.42	-0.46	-0.44	0.0008	0.0201	0.3520	0.00066	0.0066
42	GNPTAB	79158	-0.35	-0.51	-0.43	0.0037	0.0456	0.8936	0.00000	0.0000
43	DYRK2	8445	-0.38	-0.44	-0.41	0.0040	0.0472	0.5562	0.00000	0.0000
44	FAM171A1	221061	-0.47	-0.34	-0.41	0.0032	0.0423	0.6799	0.00079	0.0076
45	GAL3ST4	79690	-0.42	-0.34	-0.38	0.0033	0.0428	0.2898	0.00009	0.0013
46	C13orf18	80183	-0.46	-0.26	-0.36	0.0037	0.0456	3.0346	0.00000	0.0000
47	BRP44L	51660	0.26	0.40	0.33	0.0037	0.0456	-0.9280	0.00000	0.0000
48	DENND5A	23258	0.31	0.38	0.34	0.0036	0.0452	-0.4684	0.00275	0.0202
49	BLK	640	0.11	0.57	0.34	0.0030	0.0412	-1.0290	0.00000	0.0000
50	PCK2	5106	-0.37	1.10	0.37	0.0033	0.0428	-0.1446	0.00352	0.0245

Supplementary Figures

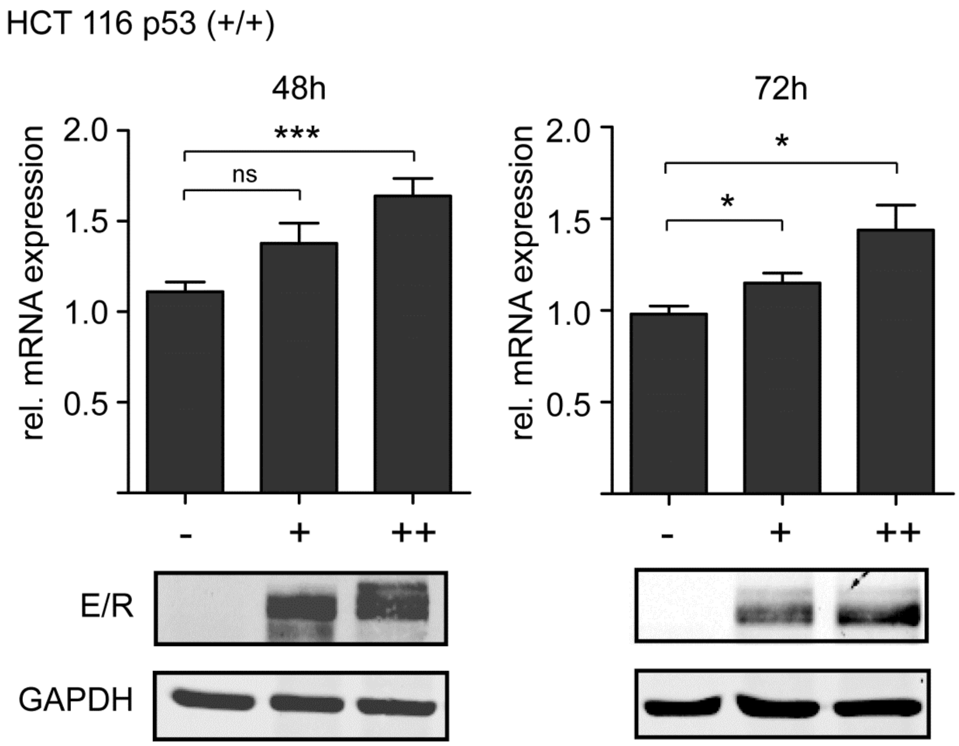

Supplementary Figure 1. MDM2 P2 transcripts upon ectopic expression of E/R in HCT116 p53$^{+/+}$ cells. Quantification of MDM2 transcripts 48h and 72h post transfection. MDM2 expression was normalized to GUS and is shown relative to the empty vector control. Depicted is pooled data of three independent experiments. Welch's t-test, *P ≤ 0.05, **P ≤ 0.01, ***P ≤ 0.001. E/R expression was confirmed by western blot analysis (bottom).

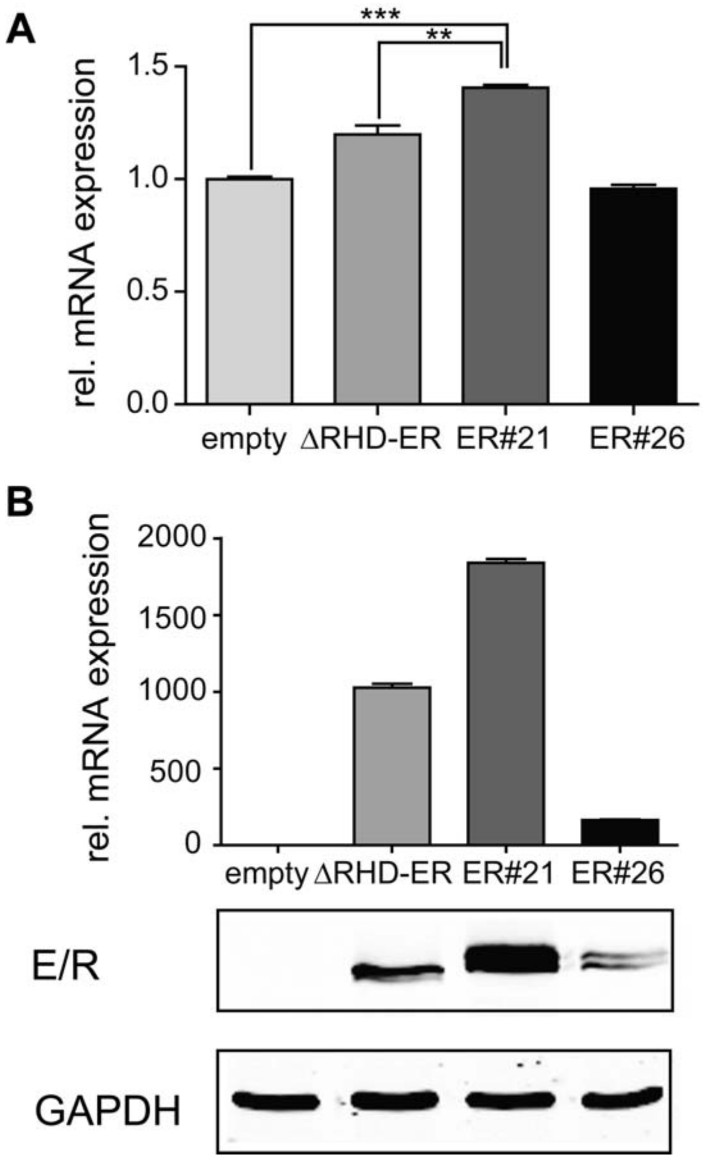

Supplementary Figure 2. Expression of MDM2 as a function of E/R. (A) Quantification of MDM2 mRNA levels in HEK 293T clones expressing different amounts of ETV6-RUNX1 (ER#21 and #26), ΔRHD-ER or the empty vector. MDM2 expression was normalized to GUS and is shown relative to the empty vector control. Welch's t-test, $**P \leq 0.005$, $***P \leq 0.0001$. (B) Quantification of E/R transcript (top) and corresponding protein levels (bottom) in ETV6/RUNX1 (ER), ΔRHD-ER and empty vector (empty) expressing HEK 293T clones. HEK 293T ER#21 expresses the fusion transcripts at an approximately 10-fold higher level than clone#26. Welch's t-test, for all: $***P \leq 0.001$

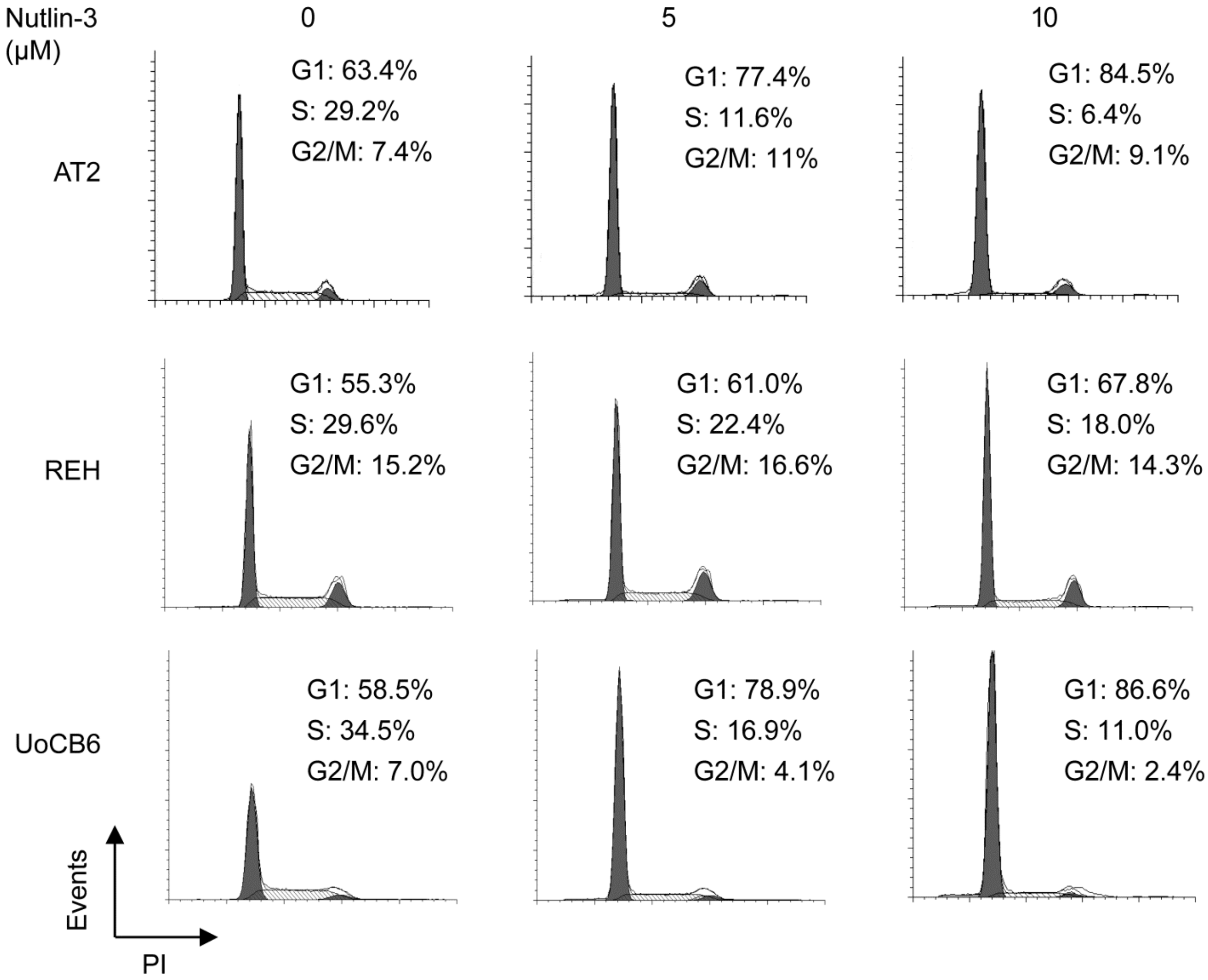

Supplementary Figure 3. Reactivation of p53 by Nutlin-3 induces cell cycle arrest in E/R-expressing leukemic cell lines. Cell cycle distribution was assessed by propidium iodide staining using BD Cycletest™ Plus-DNA Reagent Kit. Representative DNA histograms showing cell cycle profiles of three E/R-expressing cell lines upon exposure to Nutlin-3. Data were generated after 24 h exposure to Nutlin-3 at 5 and 10 μM. Welch's t-test, $*P \leq 0.05$, $**P \leq 0.01$, $***P \leq 0.001$.

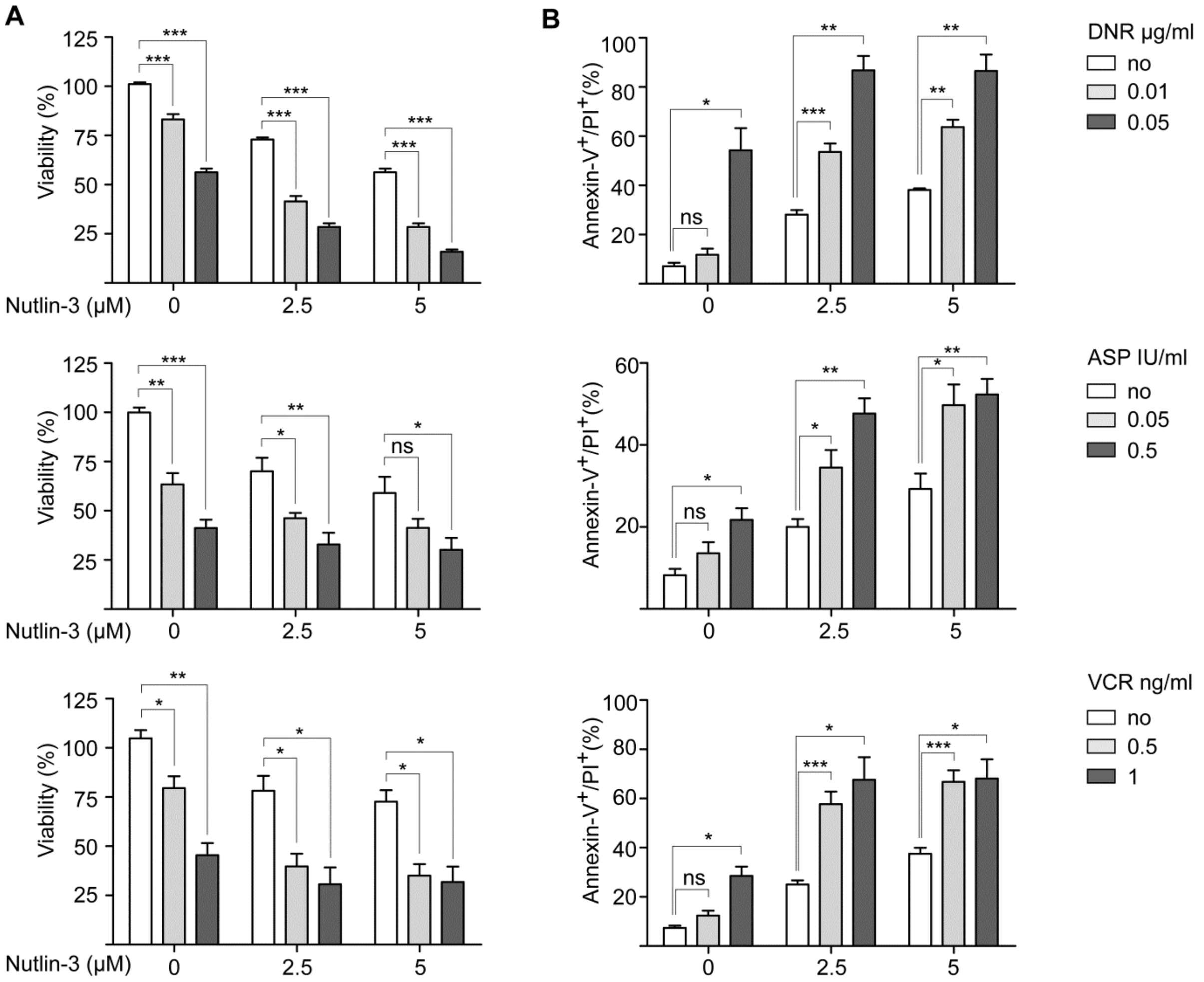

Supplementary Figure 4. Nutlin-3 enhances effects of chemotherapeutic drugs in E/R-expressing cell lines. UoCB6 cells were exposed to either daunorubicin (DNR), asparaginase (ASP) or vincristine (VCR) together with Nutlin-3. (A) Viability was determined by MTT assay and is indicated in % of carrier control. (B) Apoptotic fractions were measured by flow cytometry. The percentage of apoptotic cells (Annexin V+/PI+) is depicted. Quantification of at least three independent experiments is shown. Welch's t-test, $^{*}P \leq 0.05$, $^{**}P \leq 0.01$, $^{***}P \leq 0.001$.

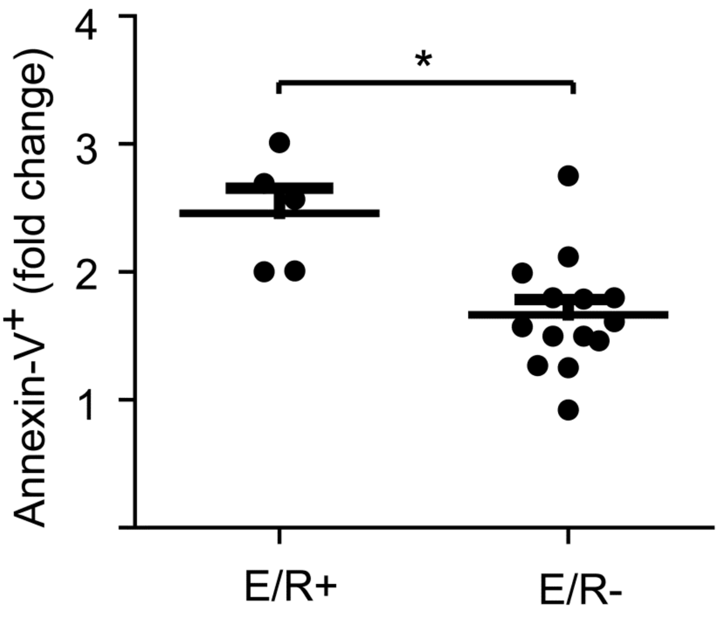

Supplementary Figure 5. Primary E/R-positive leukemic samples are more sensitive to Nutlin-3 compared to E/R-negative ones. Apoptosis levels (Annexin-V single positive cells) of E/R-positive (E/R+, n=5) and E/R-negative (E/R-, n=14) BCP-ALL samples after exposure to Nutlin-3 for 24 h are indicated as fold-changes of carrier control. Welch's t-test, *P ≤ 0.05.

1. Inthal A, Krapf G, Beck D, et al. Role of the erythropoietin receptor in ETV6/RUNX1-positive acute lymphoblastic leukemia. *Clin Cancer Res*. 2008;**14**(22):7196-7204.
2. Fuka G, Kantner HP, Grausenburger R, et al. Silencing of ETV6/RUNX1 abrogates PI3K/AKT/mTOR signaling and impairs reconstitution of leukemia in xenografts. *Leukemia : official journal of the Leukemia Society of America, Leukemia Research Fund, UK*. 2012;**26**(5):927-933.
